# Dosing and safety of amphotericin B deoxycholate in paediatric American cutaneous leishmaniasis in Peru: a case series

**DOI:** 10.1186/s12879-025-12344-w

**Published:** 2025-12-18

**Authors:** Mercedes Sanchez-Diaz, Roger Hernandez, Eduardo Verne, Jaime Gallegos, Benjamin Jordan, Joel Lopez, Elsa Gonzales Lagos, Fiorela Alvarez, Alejandro Llanos-Cuentas

**Affiliations:** 1 Unidad de Leishmaniasis y Malaria, Instituto de Medicina Tropical Alexander von Humboldt, Universidad Peruana Cayetano Heredia, Av. Honorio Delgado 430, San Martín de Porres, Lima, 15102 Peru; 2 Facultad de Medicina “Alberto Hurtado”, Universidad Peruana Cayetano Heredia, Lima, Peru; 3 Departamento de Pediatria, Hospital Cayetano Heredia, Lima, Peru; 4 Departamento de Enfermedades Infecciosas, Tropicales y Dermatológicas, Hospital Cayetano Heredia, Lima, Peru

**Keywords:** Cutaneous leishmaniasis, Amphotericin B, Paediatric, American tegumentary leishmaniasis, Neglected tropical disease

## Abstract

**Background:**

Cutaneous leishmaniasis (CL) is a parasitic disease endemic to the Americas, with a high prevalence in Peru. In resource-limited settings where liposomal amphotericin B is often unavailable, amphotericin B deoxycholate (AmB-d) remains a second-line option. However, data on its safety and effectiveness in children are scarce. This study describes the clinical experience of 20 paediatric patients with CL treated with AmB-d in a referral centre in Peru.

**Methods:**

We conducted a retrospective descriptive study of patients ≤14 years with confirmed localised CL who had failed parental sodium stibogluconate (Sb^5+^ IM/IV) treatment and received intravenous AmB-d at Hospital Cayetano Heredia (Peru) between January 2000 and December 2007. Patients received daily treatment until all lesions met the early cure criteria, defined as complete reepithelialization at the time of discharge. We collected demographic, clinical, and laboratory data, including the mean daily dose, cumulative dose, and adverse events (AEs).

**Results:**

We included 20 patients with a mean age of 4.9 years (±3.7), and 90% had lesions on the face. Nineteen patients (95%) completed treatment and achieved early clinical cure. Mean cumulative dose was 19.8 mg/kg (±5.7) and mean treatment duration was 28.8 days (±7.1). A total of 63.2% (12/19) of patients achieved clinical cure with a cumulative dose below 20mg/kg, and 84.2% with a dose below 25 mg/kg. Systemic adverse events (AEs), including fever (90%) and anorexia (70%), occurred mainly between the second and third weeks of treatment (83%). Six patients (30%) developed an increase in serum creatinine and nine (45%) developed hypokalemia.

**Conclusions:**

Intravenous amphotericin B deoxycholate (AmB-d) is an effective and well-tolerated second-line treatment for paediatric American CL unresponsive to Sb^5+^ treatment. Despite the need for high cumulative doses, prolonged treatment durations, and frequent AEs, side effects were mild to moderate, transient, and did not lead to treatment discontinuation.

**Supplementary Information:**

The online version contains supplementary material available at 10.1186/s12879-025-12344-w.

## Introduction

Cutaneous leishmaniasis (CL) is a neglected tropical disease (NTD) caused by protozoa of the *Leishmania* genus and transmitted by the bite of infected sandflies. It is endemic in 98 countries across both the New World (the Americas) and the Old World (the Middle East, Asia, Africa, and Europe) [1].

In the Americas, Peru has a significant burden of disease, reporting an estimated 5,000–11,000 cases annually, with 94% classified as CL and 6% as mucocutaneous leishmaniasis (MCL) [2, 3]. The predominant species in Peru are *Leishmania (Viannia) braziliensis* and *L. (V.) guyanensis* in the jungle, and *L. (V.) peruviana* in the Andean region [4, 5].

Leishmaniasis disproportionately affects children and young adults. The proportion of paediatric CL cases varies by country — from 2% in Paraguay to 55% in El Salvador — with an estimated prevalence of 14% in Peru estimated % 14 prevalence in [3]. This variation reflects differences in transmission settings; in domestic or peridomestic environments, children may account for up to 50% of cases, whereas in jungle regions, where exposure is primarily occupational, they represent approximately 10% [6–9].

In children, lesions frequently affect the face, increasing the risk of permanent disfiguring, scarring and social stigma; therefore, early and effective g treatment is crucial [10]. Currently, there is no standardised, universally safe, and highly effective treatment for CL in children in the Americas. Management of CL in this population is complicated by species variability, differences in clinical presentation, regional differences in drug efficacy, and a lack of paediatric data [11].

Regional treatment guidelines recommend a species-directed approach. Miltefosine, an oral agent, is a first-line option for localised CL caused by *L. braziliensis*, *L. guyanensis*, *L. panamensis*, or *L. mexicana* [12]. However, its use is limited by cost, availability, and variable efficacy, with cure rates ranging from 50 to 80% in adults [13, 14]. Miltefosine appears less effective in children under 12 years of age [15], potentially due to lower plasma drug levels when using standard weight-based dosing [16, 17], underscoring the need to optimise dosing regimens in this age group [11, 18].

Pentavalent antimonials (Sb^5**+**^) remain widely used in paediatric CL in the Americas despite reduced efficacy in children, particularly those under 5 years of age. Reported cure rates in this group range from as low as 25%, compared to 67–75% in children aged 5–14 years and over 80% in adults [11, 15, 19–22]. These age-related differences can be attributed to pharmacokinetic factors, such as reduced systemic exposure in younger children. In cases of therapeutic failure with Sb^5+^ or miltefosine, amphotericin B is the recommended second-line treatment option [12, 23]. Liposomal amphotericin B is preferred due to a better safety profile [18, 24, 25]: however, its high cost and limited availability restrict its use in many endemic regions.

Amphotericin B binds to ergosterol precursors in the parasite membrane, altering permeability and inducing cell death [18]. Comparative evidence between lipid formulations and amphotericin B deoxycholate (AmB-d) remains limited, and no clear superiority of one over the other has been demonstrated [26]. Thus, AmB-d continues to be widely used, particularly in resource-limited settings. Most published data on AmB-d for CL are from adult populations, with reported cure rates ranging from 46% to 85%, depending on the Leishmania species [27–30]. In contrast, data on its use in children are scarce, and no consensus exists on optimal dosing or safety profiles in paediatric populations [18].

This study describes the dosing and safety of amphotericin B deoxycholate (AmB-d) in a cohort of 20 paediatric patients with American CL refractory to pentavalent antimonials (Sb^5**+**^).

## Methods

### Study design

We conducted a retrospective case series study of patients aged ≤14 years with parasitologically confirmed localised CL treated with intravenous amphotericin B deoxycholate (AmB-d) between January 2000 and December 2007. Patients from various regions of Peru were treated at Hospital Cayetano Heredia in Lima, Peru, a national referral centre for leishmaniasis. The study protocol was approved by the Institutional Ethics Committee.

### Inclusion and exclusion criteria

We included patients under 14 years of age with confirmed localised CL and therapeutic failure after at least one complete cycle of parenteral (intravenous or intramuscular) pentavalent antimonials (Sb) administered at 20 mg/kg/day for 20 days. Therapeutic failure was defined as the presence of new lesions, wound reopening, or signs of activity (such as inflammation or infiltration) at the site of the original lesion during follow-up [22]. Diagnosis was confirmed by direct smear, culture, or histopathology. Patients with mucocutaneous, disseminated, or diffuse CL, or those with incomplete clinical records, were excluded.

### Data collection and clinical evaluation

Demographic, clinical, and laboratory data were retrospectively collected, including mean AmB-d daily dose, cumulative dose, treatment duration, and adverse events (AEs). Given the lack of data on standardized cumulative dosing for paediatric American CL, treatment was individualised. All patients were hospitalised and received daily intravenous AmB-d until all lesions met the clinical cure criteria, at which point therapy was discontinued. This approach determined both treatment duration and cumulative dose. All patients achieved clinical cure at discharge. Patients underwent routine monitoring with complete blood count, serum electrolytes, and renal function tests. Premedication with paracetamol and hydrocortisone was administered before each infusion to mitigate infusion-related reactions.

We defined early cure as complete reepithelialization of all lesions without signs of active inflammation [22]. Owing to the retrospective nature of the study, only early cure could be evaluated, and long-term outcomes (e.g., definitive cure at 3 months post-treatment initiation or relapse) were not assessed.

Systemic AEs included fever, anorexia, nausea, vomiting, malaise, chills, headache, diarrhoea, infusion-site phlebitis, dyspnea, exanthema, myalgia, and arthralgia. Hematologic AEs (anemia, leukopenia, thrombocytopenia) and renal AEs (increase in serum creatinine, hypokalemia, hypomagnesemia) were graded for severity using the Common Terminology Criteria for Adverse Events v3.0 (CTCAE) of the National Cancer Institute [31], a reference tool used in the national leishmaniasis program (Supplementary 1). All data were extracted from clinical records and were therefore subject to underreporting or missing data.

### Statistical analysis

Continuous variables were summarised as means with standard deviation (SD) if normally distributed, or medians with interquartile range (IQR) if not. Categorical variables were reported as absolute frequencies and percentages.

Bivariate exploratory analyses were performed to evaluate whether the frequency of adverse events (AEs) differed between two groups stratified by cumulative AmB-d dose (≤ median vs. > median). The Wilcoxon rank-sum test was used for continuous variables, and Fisher’s exact test for categorical variables. Pearson’s correlation coefficient was used to assess the linear relationship between cumulative AmB-d dose and selected laboratory parameters, specifically maximum serum creatinine and minimum serum potassium levels. Statistical significance was set at p < 0.05. Data were recorded using Microsoft Excel and analysed using Stata v10 and R software.

## Results

### Patient characteristics

We identified 28 records of paediatric patients treated with IV AmB-d for CL. Eight were excluded due to incomplete data, leaving 20 patients for analysis. The mean age was 4.9 years (± 3.7), with 75% under 9 years of age, and 65% female. Fourteen (70%) of the children came from Andean endemic areas, where the dominant species is *L. (V.) peruviana*, and 6 (30%) from jungle areas, where the dominant species is *L. (V.) braziliensis* [4, 5]. The species were not identified in this study.

Thirteen patients (65%) had multiple lesions, and 18 (90%) had facial lesions. The median disease duration was 8 months (IQR: 6.7–12.2). The median lesion size was 30 mm (IQR: 12–30 mm), and the median number of lesions was 2 (range: 1–3). Ulcerative lesions were observed in 80% of cases. 75% had received at least two previous cycles of parenteral pentavalent antimonials (Sb^5+^), but the interval to AmB-d initiation was not documented.

The initial laboratory parameters were within normal limits (mean ± SD): hematocrit 34% (± 2.6), leukocytes 7,663 cells/mm³ (± 2,543), platelets 310,000/mm³ (± 110,000), creatinine 0.4 mg/dL (± 0.09), urea 16 (± 5), serum potassium 3.9 mmol/L (± 0.3), and serum magnesium 1.8 mg/dL (± 0.2) (Supplementary 2).

### Treatment response

Nineteen patients (95%) completed AmB-d treatment. One patient was transferred to another institution during the second week of treatment for non-medical reasons and was excluded from the efficacy analysis.

**Fig. 1:**
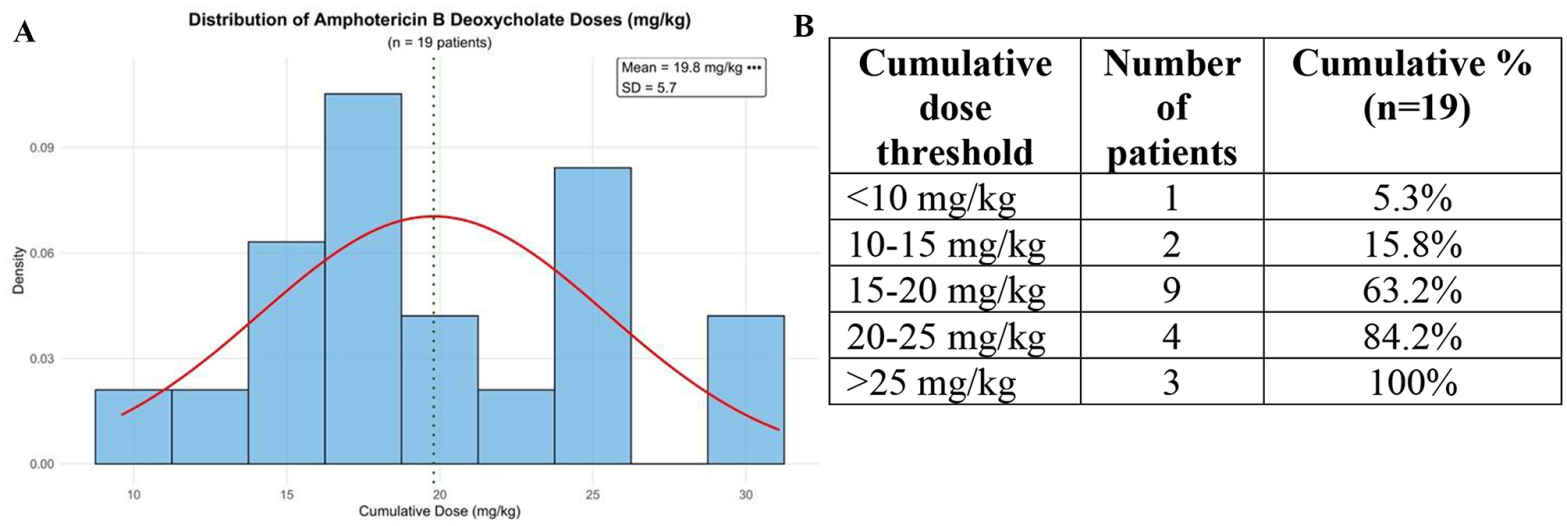
Distribution of cumulative AmB-d dose used for initial clinical cure. **(A)** Gaussian distribution of cumulative dose of AmB-d to achieve initial clinical cure. The mean dose was 19.8 mg/kg (± 5.7). **(B)** Cumulative AmB-d dose threshold for cure

The mean treatment duration was 28.8 days (± 7.1, range: 18–43 days). The mean daily dose was 0.7 mg/kg (range: 0.5–1 mg/kg), and the mean cumulative dose was 19.8 mg/kg (± 5.7, range: 9.6–31.1 mg/kg). Figure 1 shows the variation in the cumulative AmB-d dose per kg. Clinical cure was achieved in 63.2% (12/19) of patients with a cumulative dose < 20 mg/kg and 84.2% (16/19) with a cumulative dose < 25 mg/kg. The baseline characteristics and treatment response of included patients are summarised in Table 1 and Supplementary 3.

**Table 1:** Baseline characteristics and treatment response of paediatric patients with CL treated with AmB-d (*n* = 20)

Variable	Result
Demographic characteristics	
Age, mean (years; SD; range)	4.9 (± 3.7; 1-13)
Sex ratio (F:M)	1.86
Female sex, n (%)	13 (65%)
Disease duration, median (months; IQR)	8 (6.7–12.2)
Infection site, n (%)	Andean region: 14 (70%) Jungle region: 6 (30%)
Previous treatments, median (IQR)	2 (1.7-2.2)
Lesion characteristics	
Lesion number, median (range)	2 (1–3)
Lesion size, median (range, mm)	30 (10–60)
Lesion location, %	Face (90%), Upper limbs (20%), Lower limbs (5%)
Ulcerative lesions, n (%)	16/20 (80%)
Dose and duration of treatment	
Cumulative dose, mean (mg/kg; SD; range)	19.8 (± 5.7; 7.8–32.5)
Daily dose, mean (mg/kg/day; SD)	0.7 (±0.1)
Patients who completed treatment, n (%)	19/20 (95%)
Patients with initial cure at hospital discharge, n (%)	19/19 (100%)
Duration of AmB-d treatment, days (SD; range)	28.8 (± 7.1; 18–43)

Abbreviations: CL, cutaneous leishmaniasis; AmB-d, amphotericin B deoxycholate; SD, standard deviation; IQR, interquartile range; n, number; F: female; M: male

**Table 2:** Changes in creatinine levels in patients treated with IV AmB-d for CL

Changes in creatinine levels					
Patient number	Daily AmB-d dose (mg/kg/day)	Baseline creatinine (mg/dL)	Maximum creatinine (mg/dL)	Day of peak creatinine*	Cumulative AmB-d dose (mg/kg)**
4	0.7	0.56	0.89	23	15.4
5	0.6	0.59	0.75	5	2.1
7	0.3–0.6	0.52	0.79	14	8.4
11	0.7–0.8	0.40	1.1	26	14.2
12	0.7	0.50	0.80	16	11.2
15	0.5-1	0.60	1.3	10	7.3
Mean (± SD)	0.67 (± 0.05)	0.50 (± 0.07)	0.94 (± 0.21)	15.6 (± 7.8)	9.7 (± 4.9)

* Day of treatment when the highest serum creatinine level was detected

** Cumulative AmB-d dose at the time of laboratory abnormality

AmB-d: amphotericin B deoxycholate

**Fig. 2:**
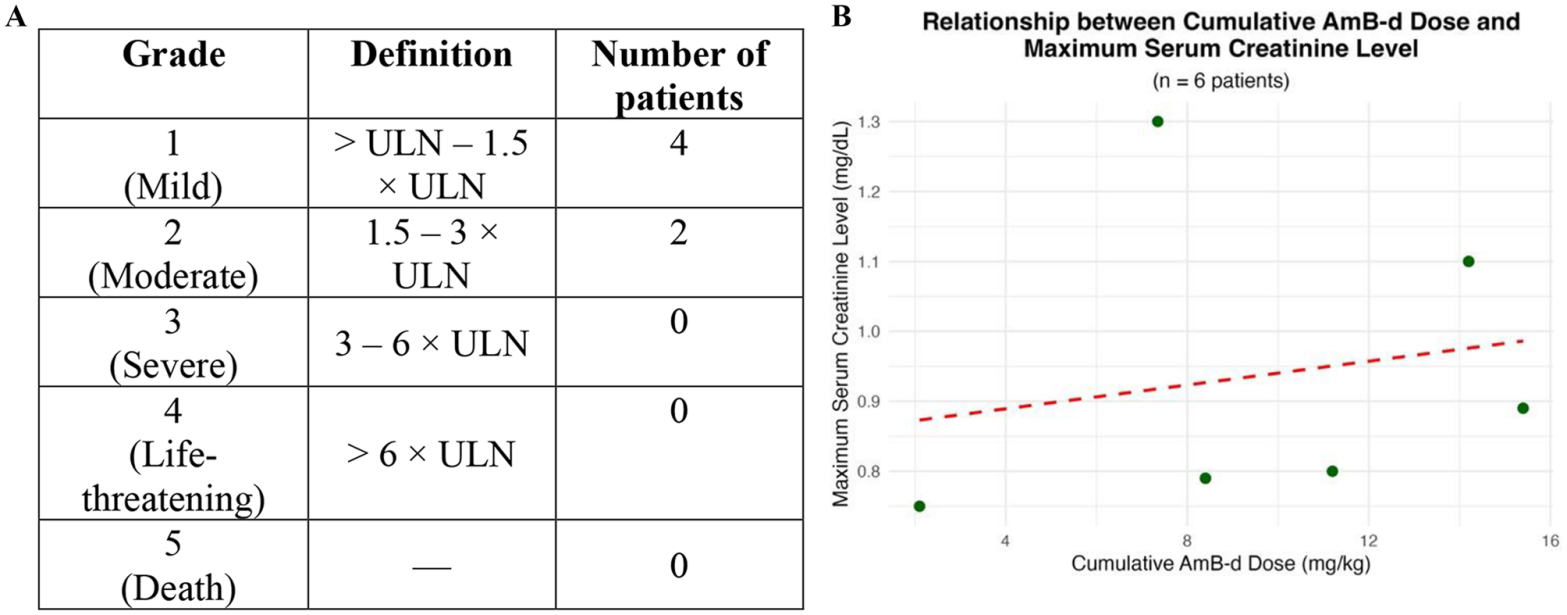
Assessment of the severity of creatinine elevation and relation to cumulative AmB-d dose. **(A)** Grading of serum creatinine elevation according to the Common Terminology Criteria for Adverse Events (CTCAE) v3.0. **(B)** Relationship between cumulative AmB-d dose and maximum creatinine level. Pearson’s correlation between variables: *r* = 0.19 (very weak or no correlation), *p* = 0.71. ULN: upper limit of normal

**Table 3:** Changes in serum potassium levels in patients treated with IV AmB-d for CL

Changes in serum potassium levels					
Patient number	Daily AmB-d dose (mg/kg/day)	Baseline potassium (mEq/L)	Minimum potassium (mEq/L)	Day of occurrence*	Cumulative AmB-d dose (mg/kg)**
1	0.6	3.7	3.2	2	1.2
3	0.6	4.6	2.7	15	9.7
5	0.6	4.2	2.8	12	6.3
6	0.5	4.4	2.5	25	12.1
11	0.7–0.8	4.3	3.2	4	2.8
15	0.5-1	3.6	2.4	10	7.3
16	1	3.9	3.4	21	20.3
18	0.8–0.9	3.8	2.5	9	6.5
20	0.7	3.7	3.3	5	3.3
Mean (± SD)	0.68 (± 0.14)	4.0 (± 0.36)	2.9 (± 0.37)	11.4 (± 7.7)	7.7 (± 5.8)

* Day of treatment when the potassium change was detected

** Cumulative AmB-d dose at the time of laboratory abnormality

AmB-d: amphotericin B deoxycholate

**Fig. 3:**
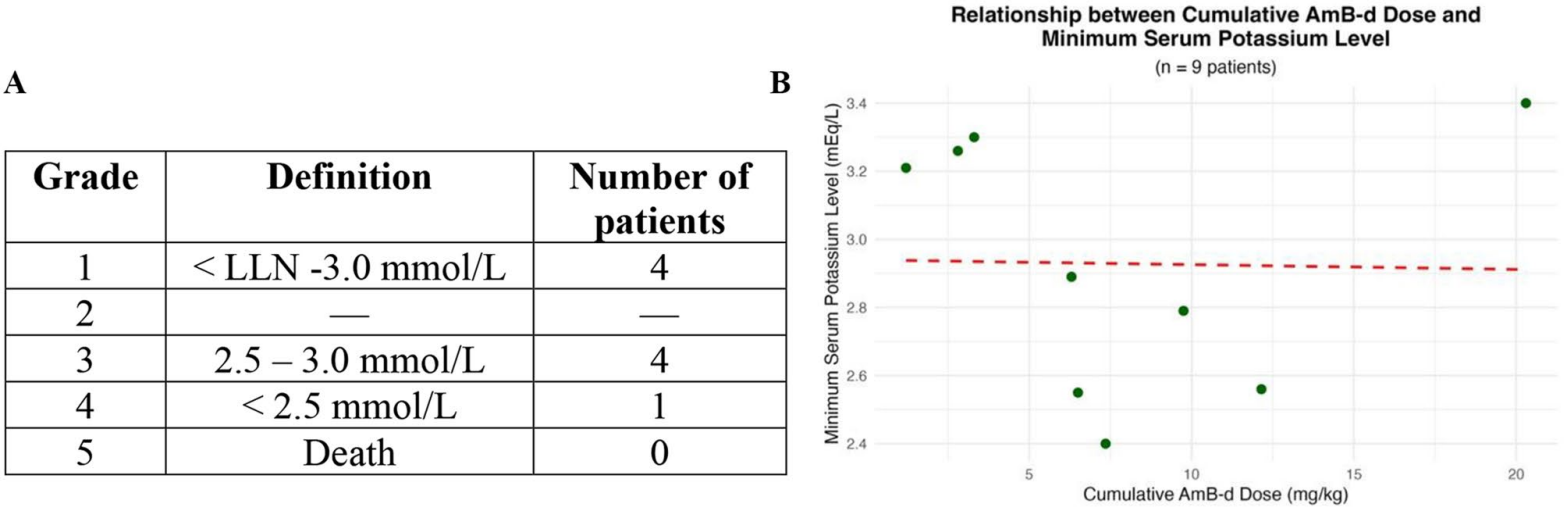
Assessment of severity of potassium decrease and its relation to cumulative AmB-d dose. **(A)** Grading of hypokalaemia according to the Common Terminology Criteria for Adverse Events (CTCAE) v3.0. **(B)** Relationship between minimum serum potassium level and cumulative Amb-d dose. Pearson’s correlation between variables: *r* = − 0.02 (no correlation), *p* = 0.956. LLN: lower limit of normal

### Safety profile

Systemic adverse events (AEs) associated with AmB-d infusion were common. Fever occurred in 18 patients (90%), followed by anorexia/hyporexia in 14 (70%), malaise in 8 (40%), nausea in 7 (35%), and chills in 7 (35%). Less frequent AEs included headache, diarrhoea, vomiting, and infusion-site phlebitis, each reported in six patients (30% each). Rash and dyspnea were observed in one patient each (5%). Four patients developed mild upper respiratory tract infections (URTIs) during hospitalization, which were self-limited and did not require antimicrobial therapy. No catheter-related bloodstream infections or other nosocomial infections were observed.

Six (30%) patients developed increases in serum creatinine levels. All were classified as grade 1 (<1.5× upper limit of normal [ULN]) or grade 2 (1.5–3× ULN) per CTCAE v3.0. In 83% of cases, elevations occurred between weeks 2 and 3 of treatment and resolved thereafter. Changes in serum creatinine level are presented in Table 2; Fig. 2.

Hypokalaemia was reported in nine patients (45%). Among them, four (44%) had grade 3 (2.5–3.0 mmol/L) and one patient (case #15) had grade 4 (< 2.5 mmol/L) hypokalaemia (Table 3; Fig. 3).

Serum magnesium levels were assessed in only nine patients (45%). Among these, one patient (case #11) developed grade 2 hypomagnesaemia (0.9–1.2 mg/dL), which coincided with an increase in serum creatinine and hypokalaemia. In this patient and in the one with grade 4 hypokalaemia (cases #11 and #15), AmB-d was temporarily suspended. Both patients received IV magnesium and potassium supplementation and resumed treatment after recovery, without further complications.

In addition, one patient who developed fever, chills, and malaise during infusion required treatment adjustment for alternate-day dosing. No patient discontinued therapy because of electrolyte abnormalities. Regarding haematological AE, two patients developed grade 2 anaemia (Hb 8–10 g/dL), which remained stable throughout AmB-d treatment and did not require intervention.

No statistically significant associations were found between age, sex, daily or cumulative AmB-d dose and the occurrence of AEs. Pearson’s correlation analysis showed no significant relationship between cumulative AmB-d dose and either maximum serum creatinine (*r* = 0.19, *p* = 0.71, Fig. 2B) or minimum serum potassium (*r* = − 0.02, *p* = 0.956, Fig. 3B).

## Discussion

This case series demonstrates that intravenous amphotericin B deoxycholate (AmB-d) is an effective and relatively safe second-line treatment for paediatric American cutaneous leishmaniasis (CL) unresponsive to pentavalent antimonials (Sb^5+^). However, its administration requires trained clinicians, supportive care, and close monitoring.

Currently, there is limited evidence regarding the optimal cumulative AmB-d dose in children. This is largely due to ethical barriers to conducting trials in the paediatric population and limited investment in neglected tropical diseases, especially in resource-limited settings. As a result, treatment regimens often rely on studies in adults and clinical judgment, which may lead to suboptimal dosing and persistent disease.

In adults, cumulative AmB-d doses of up to 25 mg/kg have been proposed for American CL [12, 18]. In our series, a mean cumulative dose of 19.8 mg/kg was observed. However, it is important to highlight that in some patients, high cumulative doses (>20 mg/kg) and prolonged treatment durations (up to 43 days) were required to achieve clinical cure. These values may help define a standard dosing regimen for paediatric use and assist in estimating length of hospitalisation and associated costs. Prolonged hospitalizations are associated with economic and psychosocial impact on families, as well as an increased risk of nosocomial infections. This need for prolonged hospitalisations is a well-recognised limitation of AmB-d, especially in resource-limited settings [11, 15, 30].

Based on our institutional experience, some of these limitations can be partially mitigated. At our national referral centre, an outpatient AmB-d protocol has been successfully implemented in adults for over a decade, with good adherence, high patient satisfaction, and reduced nosocomial infections. A similar outpatient protocol has recently been piloted in paediatric patients, with positive initial results. While in high-resource settings, liposomal amphotericin B has largely replaced AmB-d due to its superior safety and shorter regimen [30, 32], its high cost and limited availability remain major barriers in Latin America, especially in Peru [33], thus, these strategies could help improve administration of AmB-dD.

Notably, we observed considerable variability in the cumulative dose required to achieve cure, likely reflecting differences in disease chronicity and severity in our cohort. Children treated with AmB-d typically present with long-standing lesions, have failed multiple courses of systemic Sb^5+^ treatments and often have chronic and recurrent forms such as cutaneous leishmaniasis recidivans (CLR) [34, 35].

Despite these challenges, all patients achieved early clinical cure, with a response rate comparable to those reported in adult series for *L. braziliensis* (83–85%) [25, 29, 30]. Data on the efficacy of AmB-d for *L. peruviana* in adults remain limited. Although species identification was not performed, the geographic origin of our patients — both jungle (predominantly L. braziliensis) and Andean (predominantly L. peruviana) regions — suggests that both species were likely represented [4, 5].

Adverse events were common but mild to moderate, and none led to treatment discontinuation. Infusion-related systemic AEs (IRAEs), including fever, chills, malaise, and nausea, were reported in up to 90% of patients. Four patients developed mild upper respiratory tract infections (URTIs), which may have contributed to an overestimation of systemic AEs. IRAEs are attributed to activation of the Toll-like receptor 2 (TLR2) and CD14 on mononuclear cells, triggering the release of proinflammatory cytokines such as interleukin 1β, TNF-α, and IL-6 [36]. No catheter-associated or nosocomial infections were reported.

No association was found between the occurrence of IRAEs and patient age, sex, daily or cumulative dose. Despite the small sample size, this is consistent with studies in adults, which also report no clear relationships between the frequency of IRAEs and AmB-d dose parameters [37, 38]. It has been observed that the rate of administration plays a role; however, as continuous or slower infusions have been shown to reduce the prevalence and severity of IRAEs compared to conventional rapid infusion [39].

Renal toxicity was mild to moderate, transient, and occurred primarily between the second and third weeks of treatment. No significant association was found with age or daily/cumulative AmB-d dose, consistent with previous studies [29, 30]. As previously reported, children may be less susceptible to nephrotoxicity due to fewer comorbidities and intrinsic pharmacokinetic differences, such as lower volume of distribution and higher renal clearance [40].

Hypokalaemia was a frequent electrolyte disturbance, seen in 45% of patients. In contrast, hypomagnesaemia was identified in only one patient; however, this may be underestimated due to limited serum magnesium monitoring and the use of a lower diagnostic threshold (<1.2 mg/dL) compared to other studies [35]. The pathophysiology of these electrolyte abnormalities is related to the vasoconstrictive effect of AmB-d on afferent renal arterioles, which reduces renal blood flow and glomerular filtration rate [28, 36]. To prevent tubular damage, preventive measures such as adequate oral or intravenous hydration, saline loading, and adjustment of dosing intervals are recommended [37, 38].

Our study has several limitations. The retrospective design restricted access to complete clinical data, resulting in missing values for some variables. Although the study focuses on an underreported population (children with American CL), the sample size was small (20 patients), limiting the statistical power to detect associations. Additionally, species identification was not performed, precluding species-specific efficacy analysis.

Follow-up after treatment was limited, as most patients treated at our Leishmaniasis Unit are referred from rural endemic regions far from Lima, creating logistical barriers. As a result, the post-discharge follow-up rate is below 20%, and long-term outcomes, including relapse, could not be assessed. Typically, only patients with relapse return to the program. Two such cases (patients #18 and #19) relapsed one year after discharge, but others may have sought care at local facilities and thus were not captured in our records.

## Conclusions

This retrospective case series supports the use of intravenous amphotericin B deoxycholate (AmB-d) as an effective and relatively well-tolerated second-line treatment for paediatric American cutaneous leishmaniasis (CL) unresponsive to pentavalent antimonials (Sb^5**+**^). Despite the need for high cumulative doses, prolonged treatment durations, and frequent AEs, adverse events were mild to moderate, transient, and did not lead to treatment discontinuation. In resource-limited settings, where access to liposomal formulations is limited, AmB-d remains a viable and accessible alternative. Prospective studies with larger paediatric cohorts, species identification, and long-term follow-up are needed to validate these findings and guide optimal treatment strategies.

## Supplementary Information

Below is the link to the electronic supplementary material.

Supplementary Material 1

## Data Availability

The datasets generated and/or analyzed during this study are available from the corresponding author upon reasonable request.

## Abbreviations

CL: Cutaneous leishmaniasis
NTD: Neglected tropical disease
MCL: Mucocutaneous leishmaniasis
AmB-d: Amphotericin B deoxycholate
IV: Intravenous
IM: Intramuscular
Sb^5+^: Pentavalent antimonials
AE: Adverse events
IRAE: Infusion-related adverse events
SD: Standard deviation
IQR: Interquartile range
URTIs: Upper respiratory tract infections
TLR2: Toll-like receptor 2
