# Supplementary material for: Dosing and safety of amphotericin B deoxycholate in paediatric American cutaneous leishmaniasis in Peru: a case series
Source: BMC Infect Dis. 2025 Dec 18;26:120. doi: 10.1186/s12879-025-12344-w (PMC12829079; doi:10.1186/s12879-025-12344-w)
Supplement: Supplementary file 1 — Supplementary Material 1 [file 12879_2025_12344_MOESM1_ESM.docx]

**Supplementary 1**

| **Severity grading of laboratory adverse events according to CTCAE v3.0 (National Cancer Institute)** | | | | | | |
| --- | --- | --- | --- | --- | --- | --- |
| Haematologic system | | | | | | |
| **Adverse event** | **Laboratory parameter** | **Grade 1** | **Grade 2** | **Grade 3** | **Grade 4** | **Grade 5** |
| Anaemia | Haemoglobin (g/dL) | 10 - LLN | 8 – <10 | 6.5 – <8 | < 6.5 | Death |
| Leukopenia | Leukocytes  (per mm^3^) | 3000 – LLN | 2000 – <3000 | 1000 – <2000 | <1000 | Death |
| Thrombocytopenia | Platelets  (per mm^3^) | 75 000 – LLN | 50 000 – <75 000 | 25 000 – <50 000 | <25 000 | Death |

| Renal system | | | | | | |
| --- | --- | --- | --- | --- | --- | --- |
| **Adverse event** | **Laboratory parameter** | **Grade 1** | **Grade 2** | **Grade 3** | **Grade 4** | **Grade 5** |
| Renal failure | Creatinine* | ULN – 1.5 × ULN | 1.5 – 3 × ULN | 3 – 6 × ULN | > 6 × ULN | Death |
| Hypomagnesaemia | Magnesium  (mg/dL) | 1.2 – LLN | 0.9 – 1.2 | 0.7 – 0.9 | <0.7 | Death |
| Hypokalaemia | Potassium (mmol/L) | 3 – LLN | — | 2.5 – 3.0 | < 2.5 | Death |

**Supplementary 1.** Severity grading of laboratory adverse events according to CTCAE v3.0 (National Cancer Institute)

*Creatinine reference values by age from the Harriet Lane Handbook, 17th Ed.

Abbreviations: LLN: lower limit of normal; ULN: upper limit of normal

**Supplementary 2**

| **Baseline laboratory values** | | | | | | | |
| --- | --- | --- | --- | --- | --- | --- | --- |
|  | Renal | | | | Hematologic | | |
| Patient number | Creatinine  (mg/dL) | Urea  (mg/dL) | Potassium  (mmol/L) | Magnesium  (mg/dL)* | Hematocrit (%) | Leukocytes  (per mm^3^) | Platelets **(**mm^3^**)** |
| 1 | 0.44 | 8 | - | - | 32 | 7 200 | 198 000 |
| 2 | 0.46 | 12 | 3.8 | 1.4 | 33 | 4 600 | 139 000 |
| 3 | 0.52 | - | 4.6 | - | 36 | 5 600 | 356 000 |
| 4 | 0.56 | - | 4.2 | - | 35 | 5 700 | 437 000 |
| 5 | 0.59 | 10 | 4.2 | 2.1 | 30 | - |  |
| 6 | 0.38 | 16 | 4.4 | - | 35 | 4 900 | 300 000 |
| 7 | 0.52 | 13 | 4 | 1.8 | 39 | 9 800 | 450 000 |
| 8 | 0.4 | 16 | 3.8 | - | 34 | 5 600 | 230 000 |
| 9 | 0.4 | 14.9 | 4.6 | - | 30 | 13 000 | 379 000 |
| 10 | 0.3 | 10 | 3.5 | - | 35 | 10 000 | 478 000 |
| 11 | 0.4 | 21 | 4.3 | 2 | 36 | 4 600 | 206 000 |
| 12 | 0.5 | 17 | 3.9 | - | 36 | 7 700 | 362 000 |
| 13 | 0.6 | 27 | 4 | - | 39 | 6 400 | 180 000 |
| 14 | 0.3 | 17 | 3.3 | 2 | 34 | 7 700 | 206 000 |
| 15 | 0.6 | 20 | 3.6 | 1.5 | 33 | 7 800 | 460 000 |
| 16 | 0.4 | 16 | 3.9 | - | 37 | 11 200 | 190 000 |
| 17 | 0.5 | 24 | 3.9 | 2 | 31 | 6 700 | 236 000 |
| 18 | 0.3 | 18 | 3.8 | 1.8 | 33 | 7 000 | 421 000 |
| 19 | 0.4 | 22.9 | 4 | 1.9 | 31 | 7 600 | 367 000 |
| 20 | 0.4 | 20 | 3.78 | - | 37 | 12 500 | 300 000 |
| Mean (±SD) | 0.4 (±0.09) | 16 (±5) | 3.9 (±0.3) | 1.8 (±0.2) | 34 (±2.6) | 7 663  (**±**2 543) | 310 000 (**±**110 000) |

**Supplementary 2.** Baseline laboratory values

Blank spaces represent that the baseline value was not measured before AmB-d treatment initiation.

*Serum magnesium baseline and follow-up values were only measured in 9 patients.

**Supplementary 3**

| Demographic and treatment data of paediatric patients with CL treated with intravenous AmB-d (n=20) | | | | | | | | | | | | |
| --- | --- | --- | --- | --- | --- | --- | --- | --- | --- | --- | --- | --- |
| Patient No. | Age (years)/ Sex | Duration of illness*  (months) | Lesions —n | Distribution | Type(s) of lesions | Type of CL† | Maximum diameter (mm) | Previous treatments —n | Previous treatment(s) | Daily dose  (mg/kg/day) | Cumulative dose  (mg/kg) | Treatment duration (days) |
| 1 | 3/M | 6 m | 3 | Face | Ulcer, papule | Andean | 50 | 3 | Antimonials | 0.6 | 14.4 | 24 |
| 2 | 2/F | 13 m | 2 | Face | Papule, active scar | Sylvatic | 10 | 5 | Antimonials + imiquimod | 0.6 | 9.6 | 21 |
| 3 | 2/F | 5 m | 2 | Upper limbs | Ulcer, papule | Andean | 30 | 3 | Antimonials | 0.6 | 15.6 | 25 |
| 4 | 4/F | 7 m | 2 | Face | Papule, scar | Andean | 50 | 1 | Antimonials | 0.7 | 17.5 | 26 |
| 5 | 1/M | 7 m | 2 | Face | Active scar | Sylvatic | 30 | 1 | Antimonials | 0.6 | 12.3 | 22 |
| 6 | 10/F | 9 m | 1 | Lower limbs | Ulcer | Sylvatic | 50 | 2 | Antimonials | 0.5 | 16.0 | 32 |
| 7 | 11/F | 48 m | 1 | Face | Ulcer, | Andean | 30 | 1 | Antimonials | 0.6 | 18.1 | 43 |
| 8 | 9/F | 8 m | 3 | Face, upper limbs | Ulcer, | Andean | 25 | 2 | Antimonials | 0.7 | 16.8 | 24 |
| 9 | 10 m/m | 8 m | 3 | Face | Ulcer, scar | Sylvatic | 15 | 1 | Antimonials | 1.0 | 28.8 | 30 |
| 10 | 3/M | 7 m | 1 | Face | Papule-nodule | Andean | 60 | 2 | Antimonials | 0.6 | 17.1 | 29 |
| 11 | 4/F | 8 m | 1 | Face | Ulcer | Andean | 20 | 1 | Antimonials | 0.8 | 31.1 | 43 |
| 12 | 12/F | 8 m | 3 | Face, upper limbs | Ulcer, scar | Andean | ND | 4 | Antimonials | 0.7 | 18.9 | 27 |
| 13 | 3/F | 11 m | 2 | Face | Ulcer | Sylvatic | 5 | 2 | Antimonials | 1.0 | 24.2 | 18 |
| 14 | 4/M | 15 m | 1 | Face | Ulcer | Andean | 30 | 2 | Antimonials | 1.0 | 24.8 | 26 |
| 15 | 13/F | 13 m | 3 | Face, upper limbs | Ulcer, scar | Andean | 30 | 2 | Antimonials | 0.5 | 19.8 | 41 |
| 16 | 3/F | 6 m | 2 | Face | Ulcer | Andean | 10 | 2 | Antimonials, paromomicine, imiquimod | 1.0 | 23.3 | 24 |
| 17 | 2/M | 19 m | 1 | Face | Ulcer | Andean | 10 | 3 | Antimonials | 0.6 | 17.4 | 29 |
| 18 | 5/F | 6 m | 1 | Face | Ulcer | Andean | 30 | 2 | Antimonials, paromomicine, imiquimod | 0.9 | 25.0 | 31 |
| 19 | 3/M | 5 m | 2 | Face | Ulcer, scar | Andean | 10 | 2 | Antimonials | 0.8 | 25.5 | 34 |
| 20 | 5/F | 12 m | 3 | Face | Ulcer, papule | Sylvatic | 20 | 2 | Antimonials | 0.7 | ‡ | ‡ |
| Mean (±SD) / Median [IQR] ** | **4,9 years (±3.7)** | **8 m**  **[6.7-12.2]** | **2**  **[1-3]** | **-** | **-** | **-** | **30 mm [12.5-30]** | **2**  **[1.7-2.2]** | **-** | **0.7**  **(±0.1)** | **19,8 mg/kg**  **(±5,7)** | **28,8 days (±7,1)** |

**Supplementary 3.** Demographic and treatment data of paediatric patients with CL treated with intravenous deoxycholate amphotericin B

*Duration of illness at the time of hospital admission for amphotericin treatment.

**Mean with standard deviation (SD) was reported for normally distributed continuous data. Median with interquartile range (IQR) was used for skewed or discrete data.

†Andean/Sylvatic CL was defined based on the probable region where the infection was acquired.

‡The patient was transferred to another hospital in the second week of treatment for non-medical reasons but had shown signs of improvement with therapy.

Abbreviations: m, months; CL, cutaneous leishmaniasis; AmB-d, amphotericin B deoxycholate; ND, no data.
